# Impact of preoperative shoulder osteoarthritis severity score on outcomes after rotator cuff repair: A correlation study

**DOI:** 10.1038/s41598-026-58506-x

**Published:** 2026-06-19

**Authors:** Peerapat Lertwiram, Praman Fuangfa, Chusak Kijkunasathian, Nadhaporn Saengpetch, Chalermchai Limitlaohaphan, Chatchawan Lertbutsayanukul, Chaiyanun Vijittrakarnrung

**Affiliations:** 1 Department of Orthopedics, Faculty of Medicine Ramathibodi Hospital, Mahidol University, Bangkok, Thailand; 2 Department of Diagnostic Radiology, Faculty of Medicine Ramathibodi Hospital, Mahidol University, Bangkok, Thailand

**Keywords:** Arthroscopic rotator cuff repair, Glenohumeral osteoarthritis, Patient-Reported outcomes, Shoulder osteoarthritis severity score, Rotator cuff tear, Diseases, Medical research, Rheumatology, Risk factors

## Abstract

Arthroscopic rotator cuff repair (ARCR) generally improves clinical outcomes, but the impact of pre-existing glenohumeral osteoarthritis (GHOA) severity on these outcomes remains unclear. This study aims to evaluate the correlation between preoperative GHOA severity, assessed via the Shoulder Osteoarthritis Severity (SOAS) score, and postoperative patient-reported outcomes (PROs) following ARCR with a minimum of two years follow-up. In this retrospective cohort study, 150 patients who underwent ARCR between 2018 and 2023 were included. Preoperative GHOA severity was assessed independently by two reviewers using magnetic resonance imaging (MRI) and graded using the SOAS score. PROs included the American Shoulder and Elbow Surgeons (ASES) score, University of California-Los Angeles (UCLA) score, and Western Ontario Rotator Cuff (WORC) index. Correlation and multivariate regression analyses were used to identify predictors of PROs. Receiver operating characteristic (ROC) curve was performed to assess the SOAS score’s predictive value. Of 150 eligible patients, the cohort had a mean age of 66.6 ± 8.8 years and a mean follow-up of 42.3 ± 14.5 months. Inter-reader reliability of total SOAS was excellent (ICC = 0.942). Higher total SOAS scores were significantly associated with lower ASES (*r*=–0.21, *p* = 0.012) and UCLA (*r*=–0.22, *p* = 0.006) scores. Subdomain analysis revealed that labral-bicipital complex pathology negatively correlated with all three PROs (ASES: *r*=-0.21, *p* = 0.010; UCLA: *r*=-0.20, *p* = 0.013; WORC: *r*=-0.18, *p* = 0.026), while cartilage degeneration correlated with lower UCLA score (*r*=-0.18, *p* = 0.033). On multivariate analysis, long head of biceps pathology was an independent predictor of a lower ASES score (β=–2.14, *p* = 0.029) and WORC index (β=–0.1, *p* = 0.032). An optimal SOAS cut-point of 17 for predicting failure showed poor discriminative ability (AUC = 0.596). The SOAS score demonstrated a significant correlation with Kellgren-Lawrence grading system (*r* = 0.71, *p* < 0.001). Although continuous GHOA severity correlates with lower postoperative scores, the overall SOAS score alone is a poor prognostic discriminator for clinical failure. Therefore, GHOA should not be considered an absolute contraindication for ARCR. A holistic preoperative assessment integrating these critical factors is essential for optimizing clinical decision making.

## Introduction

 Rotator cuff injuries are a prevalent clinical problem, significantly impacting patients’ quality of life through shoulder pain, functional limitations, and reduced work productivity. While conservative management, including analgesics and physical therapy, is often the initial approach, a substantial proportion of patients ultimately require surgical intervention in the form of rotator cuff repair. Surgical intervention, in the form of arthroscopic rotator cuff repair (ARCR), aims to restore shoulder strength, range of motion, and overall function, thereby enabling patients to return to their desired activities. An estimated 250,000 to 300,000 ARCRs are performed annually in the United States^1,2^. The increasing prevalence of this surgery has prompted extensive research into identifying factors that may negatively influence postoperative outcomes. These factors include patient-specific characteristics such as sex^3,4^, age^5,6^, smoking status^7,8^, occupation^3,9^, and body mass index^10,11^, as well as injury-related factors like tear size^12,13^ and the presence of fatty infiltration^14,15^. Identifying these risk factors is crucial for optimizing patient selection, tailoring surgical techniques, and implementing targeted rehabilitation strategies to maximize the likelihood of successful ARCR.

Glenohumeral osteoarthritis (GHOA), a common cause of shoulder pain and functional decline in older adults, emerges as a potentially critical, yet incompletely understood, factor influencing outcomes following rotator cuff repair. Rotator cuff tears frequently coexist with GHOA ranging from 12.5% to 28%^16,17^. Managing patients with both conditions presents unique challenges, requiring careful consideration of surgical options, such as the choice between repair and replacement, and tailored rehabilitation protocols. Existing literature presents conflicting evidence regarding the impact of pre-existing GHOA on the success of ARCR, with some studies demonstrating poorer functional outcomes in patients with osteoarthritis, while others report no significant differences compared to those without the condition. This inconsistency highlights the need for more refined diagnostic approaches and a deeper understanding of the complex interplay between rotator cuff pathology and underlying glenohumeral joint degeneration.

The recent introduction of MRI-based assessment tools, such as the Shoulder Osteoarthritis Severity (SOAS) score, offers a more comprehensive evaluation of joint degeneration by assessing cartilage integrity, bone morphology, and soft tissue structures. The SOAS score has demonstrated good reliability and validity in previous studies^18^, providing a more nuanced assessment of osteoarthritis severity compared to traditional radiographic grading systems. By utilizing the magnetic resonance imaging (MRI) -based SOAS score and analyzing potential confounding variables, this study aims to provide a more accurate assessment of the impact of GHOA on ARCR outcomes. This will ultimately enable clinicians to better predict postoperative results and personalize treatment strategies for patients undergoing ARCR.

## Materials and methods

### Patient selection and study design

This study employed a retrospective cohort design and was conducted within the Department of Orthopaedics, Faculty of Medicine Ramathibodi Hospital, Mahidol University. Participants included patients diagnosed with rotator cuff tears by history taking, physical examination and MRI who underwent ARCR. Eligible patients were those aged 18 years or older with a documented rotator cuff tear, having undergone arthroscopic repair, and possessing a shoulder MRI performed within one year prior to the surgical procedure, with a minimum follow-up duration of one year. Patients were excluded if they had undergone revision ARCR, an inability to achieve anatomic repair, exhibited fatty infiltration graded as Goutallier grade 3 or 4, or were unable to communicate or complete questionnaires. This study was fully approved by the Ramathibodi Hospital Institutional Review Board (COA. MURA2024/586). All participants provided written informed consent.

### Data collection

Patients with rotator cuff tears undergoing ARCR were identified and recruited from January 2018 to July 2023. The surgical procedures were performed by five fellowship-trained sports medicine orthopaedic surgeons. Data were collected from electronic medical records, including demographics, medical history, pre-operative images, surgical details, and post-operative complications. Standardized telephone interview was conducted at least two year post-operatively to collect patient-reported outcome measures (PROs) including the American Shoulder and Elbow Surgeons (ASES) score, University of California-Los Angeles (UCLA) score, and Western Ontario Rotator Cuff (WORC) index.

A comprehensive assessment was performed, encompassing patient-specific factors, including age, sex, dominant shoulder, occupation, comorbidities (e.g., diabetes mellitus (DM), osteoporosis), smoking history, prior trauma, and body mass index (BMI), as well as surgical factors, such as ARCR technique (single row, double row suture bridge repair), acromioplasty, long head biceps management (biceps tenotomy, tenodesis). These factors were collected alongside rotator cuff tear characteristics (tear size, tear type, retraction, fatty infiltration), long head biceps tendon pathology (lesion and instability), glenohumeral arthritis (assessed by SOAS score, Samilson and The Kellgren and Lawrence (KL) grading), and prior upper extremity procedures to identify potential confounders influencing patient-reported outcomes.

GHOA was assessed using pre-operative shoulder MRI, using a magnet strength of at least 1.5T with axial, coronal, and sagittal fat-suppressed intermediate-weighted images, and coronal and sagittal T1-weighted sequences. Glenohumeral osteoarthritis was quantified using the SOAS) score. The SOAS score is a composite measure encompassing several key components: Rotator cuff integrity (0–35 points), considering tear size, retraction, muscle fatty infiltration, and muscle atrophy; the Labral-bicipital complex (0–12 points), evaluating the glenoid labrum, para-labral ganglia, long head biceps tendon, and glenohumeral ligaments; Cartilage (0–12 points), based on sub-scores in the adjusted Whole-Organ Magnetic Resonance Imaging Score; Osseous structure (0–24 points), assessing bone marrow edema, intra-osseous cysts, osteophytes, and bone deformity; the Joint capsule (0–9 points), evaluating synovitis/obliteration, joint effusion, and loose bodies; and the Acromion (0–8 points), considering bursitis, acromioclavicular joint degeneration, and acromion deformity^18^. SOAS scores were independently assessed by two reviewers: a musculoskeletal radiologist and a sports medicine orthopaedic surgeon. The SOAS score threshold of 18 points was selected based on which demonstrated that this cutoff provided optimal sensitivity and specificity for detecting clinically significant glenohumeral osteoarthritis^18^. The resulting SOAS scores from the two reviewers will be used to assess inter-reader reliability. Prior to commencing the actual MRI analysis, the reviewers will participate in a training session using MRI images from five patients not included in the study to standardize the interpretation of the SOAS score and ensure consistency in the research process.

In addition to MRI, plain radiographs, including shoulder true anteroposterior (AP) and supraspinatus outlet views, were obtained to assess glenohumeral osteoarthritis using the Samilson-Prieto and KL grading systems. The Samilson grading was defined as follows: Grade 1 (Mild): Osteophytes less than 3 mm in height; Grade 2 (Moderate): Osteophytes between 3 and 7 mm in height; Grade 3 (Severe): Osteophytes greater than 7 mm in height. The KL grading was defined as follows: Grade 1: Possible joint space narrowing and osteophyte formation; Grade 2: Definite osteophyte formation with possible joint space narrowing; Grade 3: Multiple osteophytes, definite joint space narrowing, sclerosis, and possible bony deformity; Grade 4: Large osteophytes, obliterated joint space narrowing, severe sclerosis, and definite bony deformity. Radiographic and MRI-based grading were correlated to validate the SOAS score’s clinical utility.

The primary outcome measure was the American Shoulder and Elbow Surgeons (ASES) score^19^. Secondary outcome measures included the University of California-Los Angeles (UCLA) score^20^ and the Western Ontario Rotator Cuff (WORC) index^21^. The validated Thai versions of these questionnaires were administered to all participants.

### Statistical analysis

Patient characteristics and other outcomes are summarized using means with standard deviations for continuous variables, and frequencies with percentages for categorical variables. For baseline data, continuous variables were compared between groups using independent t-tests and Mann-Whitney U test for normally and not normally distributed data, respectively, while categorical variables were assessed using either Chi-square or Fisher’s exact tests, as appropriate. The correlation between SOAS scores (including sub-domains) and postoperative patient-reported outcomes (ASES, UCLA, and WORC) was evaluated. Pearson correlation coefficients were utilized for normally distributed continuous variables, and Spearman’s rank correlation was applied for ordinal variables or non-normally distributed data. To identify independent predictors of clinical outcomes, multiple linear regression analysis was performed. Factors demonstrating a correlation with *p* < 0.10 in the univariate analysis were entered into the multivariate models. To address potential multicollinearity between the total SOAS score and its constitutive sub-domains, we constructed two distinct multivariate models. Model 1 incorporated individual SOAS sub-domains along with potential confounders. Model 2 focused on the total SOAS score as a single independent variable alongside the same confounders. To determine the optimal SOAS score cut-off for predicting surgical outcomes, a receiver operating characteristic (ROC) curve analysis was performed. Clinical success was defined by achieving the Patient Acceptable Symptom State (PASS) for the ASES score. Based on previously established literature for arthroscopic rotator cuff repair, an ASES score of ≥ 86.7 was utilized as the PASS threshold^9^. Furthermore, the validity of the SOAS score was assessed by examining its correlation with traditional radiographic grading systems, including the Kellgren-Lawrence and Samilson-Prieto classifications. Inter-reader reliability for the SOAS score was assessed using the Intraclass Correlation Coefficient (ICC). All statistical analyses were performed using STATA 18.0 (StataCorp, College Station, Texas, USA), with significance defined as *p* < 0.05.

### Sample size calculation

The sample size was estimated using a formula for comparing means between two independent samples, with the following specifications: Alpha error was set at 0.05, and the power of the study was set at 0.8. The alternate hypothesis assumed a difference in mean ASES scores between the groups with and without glenohumeral osteoarthritis equal to the Minimally Clinically Important Difference (MCID) of 11.10^9^. The standard deviation of ASES scores in the group with glenohumeral osteoarthritis was 23.00^22^, while the standard deviation of ASES scores in the group without glenohumeral osteoarthritis was 22.41^22^. The ratio between the groups with and without glenohumeral osteoarthritis (r) was 1.0. This calculation yielded a total required sample size of 138 participants. This study ultimately recruited 150 patients.

## Results

A total of 150 patients were included in this study, with a mean age of 66.62 ± 8.79 years. The majority of the cohort consisted of female patients (57.33%). Comorbidities included DM in 19.33% and osteoporosis in 6.67% of the participants. The mean postoperative ASES, UCLA, and WORC scores for the entire population were 94.86 ± 9.06, 33.55 ± 2.46, and 98.8 ± 0.41, respectively. Detailed baseline demographics and clinical outcomes are presented in Table 1. The inter-reader reliability for the total SOAS score was excellent, with an Intraclass Correlation Coefficient (ICC) of 0.942 (95% CI [0.899, 0.966]). Reliability for sub-domains ranged from 0.619 for cartilage to 0.912 for the acromion, as shown in Table 2.

**Table 1:** Baseline characteristics of the study population (*N* = 150).

Variables	Value
Demographic data	
Age (years), mean (SD)	66.62 (8.79)
Male (%)	64 (42.67)
BMI (kg/m2), mean (SD)	25.56 (4.13)
Diabetes Mellitus (%)	29 (19.33)
Osteoporosis (%)	10 (6.67)
Smoking (%)	5 (3.33)
Etiology: trauma (%)	42 (28.00)
Follow up (months), mean (SD)	42.28 (14.53)
Patient reported outcome	
ASES score, mean (SD)	94.86 (9.06)
UCLA score, mean (SD)	33.55 (2.46)
WORC index, mean (SD)	98.8 (0.41)
Intra-operative finding and procedure	
Tear size (mm.), mean (SD) (antero-posterior diameter)	14.39 (6.91)
Subscapularis tear (%)	82 (54.67)
Biceps tendinitis (%)	87 (58.00)
Subacromial impingement (%)	112 (74.67)
Repair technique (%) - Single row - Double row	50 (33.33) 100 (66.67)

**Table 2:** Interclass correlation.

SOAS score	Value	ICC (95%CI)
Rotator cuff, median (IQR)	6 (4, 9)	0.906 (0.838, 0.945)
Labral-bicipital complex, median (IQR)	2 (2, 3)	0.759 (0.585, 0.860)
Cartilage, median (IQR)	3 (2, 4)	0.619 (0.343, 0.779)
Osseous findings, median (IQR)	2 (1, 4)	0.725 (0.526, 0.840)
Joint capsule, median (IQR)	1 (1, 2)	0.749 (0.567, 0.854)
Acromion, mean (SD)	1.89 (0.89)	0.912 (0.849, 0.949)
Total score, mean (SD)	18.69 (7.54)	0.942 (0.899, 0.966)

Pearson and Spearman correlation analyses demonstrated that the total SOAS score was negatively associated with ASES (*r* = −0.205, *p* = 0.012) and UCLA (*r* = −0.224, *p* = 0.006) scores. Among the sub-domains, the labral-bicipital complex, specifically long head of the biceps pathology, showed consistent negative correlations with all three patient-reported outcomes (ASES: *r* = −0.221, *p* = 0.007; UCLA: *r* = −0.2, *p* = 0.015; WORC: *r* = −0.177, *p* = 0.031), while cartilage degeneration correlated with lower UCLA score (*r* = −0.175, *p* = 0.033). Systemic factors, including diabetes mellitus and osteoporosis, also significantly correlated with poorer outcomes across all metrics **(**Table 3**)**.

**Table 3:** Correlation Analysis Between Patient Factors, SOAS Sub-domains, and Postoperative Patient-Reported Outcomes.

Comparison	ASES score		UCLA score		WORC index	
	*r*	*P*-value	*r*	*P*-value	*r*	*P*-value
Patient characteristics						
Age	−0.078	0.342	−0.171	0.036*	−0.138	0.092
Male	−0.112	0.173	−0.119	0.146	−0.242	0.003*
BMI	−0.148	0.07	−0.14	0.087	−0.118	0.152
DM	−0.326	< 0.001*	−0.255	0.002*	−0.37	< 0.001*
Osteoporosis	−0.195	0.024*	−0.192	0.025*	−0.242	0.005*
Smoking	0.049	0.604	0.008	0.97	0.003	0.97
Trauma	0.006	0.95	−0.071	0.39	−0.039	0.633
SOAS score						
Total SOAS score	−0.205	0.012*	−0.224	0.006*	−0.137	0.094
1. Rotator cuff	−0.071	0.31	−0.085	0.3	−0.124	0.13
Supraspinatus/infraspinatus tear	−0.09	0.273	−0.087	0.293	−0.056	0.497
Subscapularis tear	−0.119	0.148	−0.088	0.286	−0.047	0.567
Retraction	−0.034	0.677	−0.032	0.695	−0.061	0.462
Fatty infiltration	−0.008	0.922	−0.031	0.707	−0.092	0.264
Atrophy	−0.058	0.479	−0.12	0.147	−0.092	0.267
2. Labral-bicipital complex	−0.213	0.01*	−0.203	0.013*	−0.182	0.026*
Labrum	−0.143	0.081	−0.154	0.06	−0.127	0.123
Paralabral ganglia	−0.003	0.893	0.033	0.768	0.021	0.892
Long head of the biceps	−0.221	0.007*	−0.2	0.015*	−0.177	0.031*
Glenohumeral ligament	−0.042	0.608	−0.048	0.559	−0.065	0.431
3. Cartilage	−0.09	0.272	−0.175	0.033*	−0.085	0.302
Cartilage	−0.091	0.268	−0.176	0.032*	−0.086	0.295
4. Osseous findings	−0.07	0.395	−0.069	0.401	−0.022	0.793
Bone marrow edema	0.007	0.944	0.019	0.829	0.079	0.341
Intraosseous cyst	−0.046	0.572	−0.081	0.328	−0.082	0.318
Osteophyte	−0.072	0.385	−0.042	0.61	0.013	0.875
Bone deformity	−0.022	0.786	−0.043	0.6	−0.044	0.588
5. Joint capsule	0.051	0.534	0.104	0.206	0.021	0.8
Synovitis	0.068	0.41	0.116	0.158	0.04	0.631
Joint effusion	−0.04	0.625	−0.015	0.848	−0.031	0.7
6. Acromion	−0.154	0.059	−0.11	0.178	−0.121	0.139
Bursa	−0.009	0.91	0.013	0.879	−0.057	0.493
AC joint degeneration	−0.116	0.158	−0.172	0.036*	−0.075	0.36

Two multivariate linear regression models were utilized to identify independent predictors. Model 1 (Sub-domains), DM (β = −4.9 ± 1.80, *p* = 0.007) and long head of the biceps pathology (β = −2.14 ± 0.96, *p* = 0.028) were identified as significant independent predictors of lower ASES scores **(**Table 4A**)**. Osteoporosis was a strong independent predictor for lower UCLA (β = −3.19 ± 0.73, *p* < 0.001) and WORC (β = −0.53 ± 0.12, *p* < 0.001) scores. Model 2 (Total Score), the total SOAS score remained an independent predictor for ASES (β = −0.24 ± 0.09, *p* = 0.011) and UCLA (β = −0.06 ± 0.03, *p* = 0.023) scores after adjusting for diabetes and BMI **(**Table 4B**)**.

**Table 4:** A: Multivariate analysis of SOAS sub-domains and confounders (Model 1).

ASES score			
Variable	Coefficient	SE	*P*-value
BMI	−0.18	0.17	0.294
DM	−4.9	1.8	0.007*
Osteoporosis	−5.18	2.8	0.067
Long head of the biceps	−2.14	0.96	0.028*
Labrum	−1.46	1.17	0.213

**Table 4:** B: Multivariate analysis of total SOAS Score and Confounders (Model 2).

Variable	ASES score			UCLA score		
	Coefficient	SE	*P*-value	Coefficient	SE	*P*-value
BMI	−0.2	0.17	0.251	−0.06	0.04	0.196
DM	−5.84	1.79	0.001*	−1.31	0.47	0.005*
Osteoporosis	−5.18	2.8	0.066	−3.1	0.73	< 0.001*
Total SOAS score	−0.24	0.09	0.011*	−0.06	0.03	0.023*

The SOAS score threshold of 18 points was selected based on a previous study^18^, which demonstrated that this cutoff provides optimal sensitivity and specificity for detecting clinically significant glenohumeral osteoarthritis. Patients were stratified into GHOA (SOAS ≥ 18, *n* = 75) and No GHOA (SOAS < 18, *n* = 75) groups. The GHOA group was significantly older (69.09 ± 7.83 vs. 64.15 ± 9.05 years; *p* < 0.001) and exhibited significantly larger tear sizes (17.17 ± 7.30 vs. 11.60 ± 5.19 mm; *p* < 0.001). Biceps tendinitis was significantly more prevalent in the GHOA group (77.33% vs. 38.67%; *p* < 0.001). Regarding clinical outcomes, the GHOA group had a significantly lower mean ASES score compared to the no GHOA group (93.38 ± 10.45 vs. 96.33 ± 7.18; *p* = 0.046) (Table 5).

**Table 5:** Comparison of patient characteristics and clinical outcomes between groups.

Variables	SOAS score < 18	SOAS score ≥ 18	*P*-value
Demographic data			
Age (years), mean (SD)	64.15 (9.05)	69.09 (7.83)	< 0.001*
Male (%)	30 (40.00)	34 (45.33)	0.509
BMI (kg/m2), mean (SD)	25.05 (4.10)	26.06 (4.12)	0.136
Diabetes Mellitus (%)	14 (18.67)	15 (20.00)	0.836
Osteoporosis (%)	5 (6.67)	5 (6.67)	1
Smoking (%)	3 (4.00)	2 (2.67)	1
Etiology: trauma (%)	18 (24.00)	24 (32.00)	0.275
Follow up (months), mean (SD)	42.63 (14.81)	41.93 (14.33)	0.771
Patient reported outcome			
ASES score, mean (SD)	96.33 (7.18)	93.38 (10.45)	0.046*
UCLA score, mean (SD)	33.93 (2.13)	33.17 (2.71)	0.057
WORC index, mean (SD)	98.86 (0.37)	98.75 (0.45)	0.084
Intra-operative finding and Procedure			
Tear size (mm.), mean (SD)	11.60 (5.19)	17.17 (7.30)	< 0.001*
Biceps tendinitis (%)	29 (38.67)	58 (77.33)	< 0.001*
Subacromial impingement (%)	60 (20.00)	52 (69.33)	0.133
Acromioplasty (%)	61 (81.33)	52 (69.33)	0.089
Repair technique (%) - Single row - Double row	37 (49.33) 38 (50.67)	13 (17.33) 62 (82.67)	< 0.001*

**Fig. 1:**
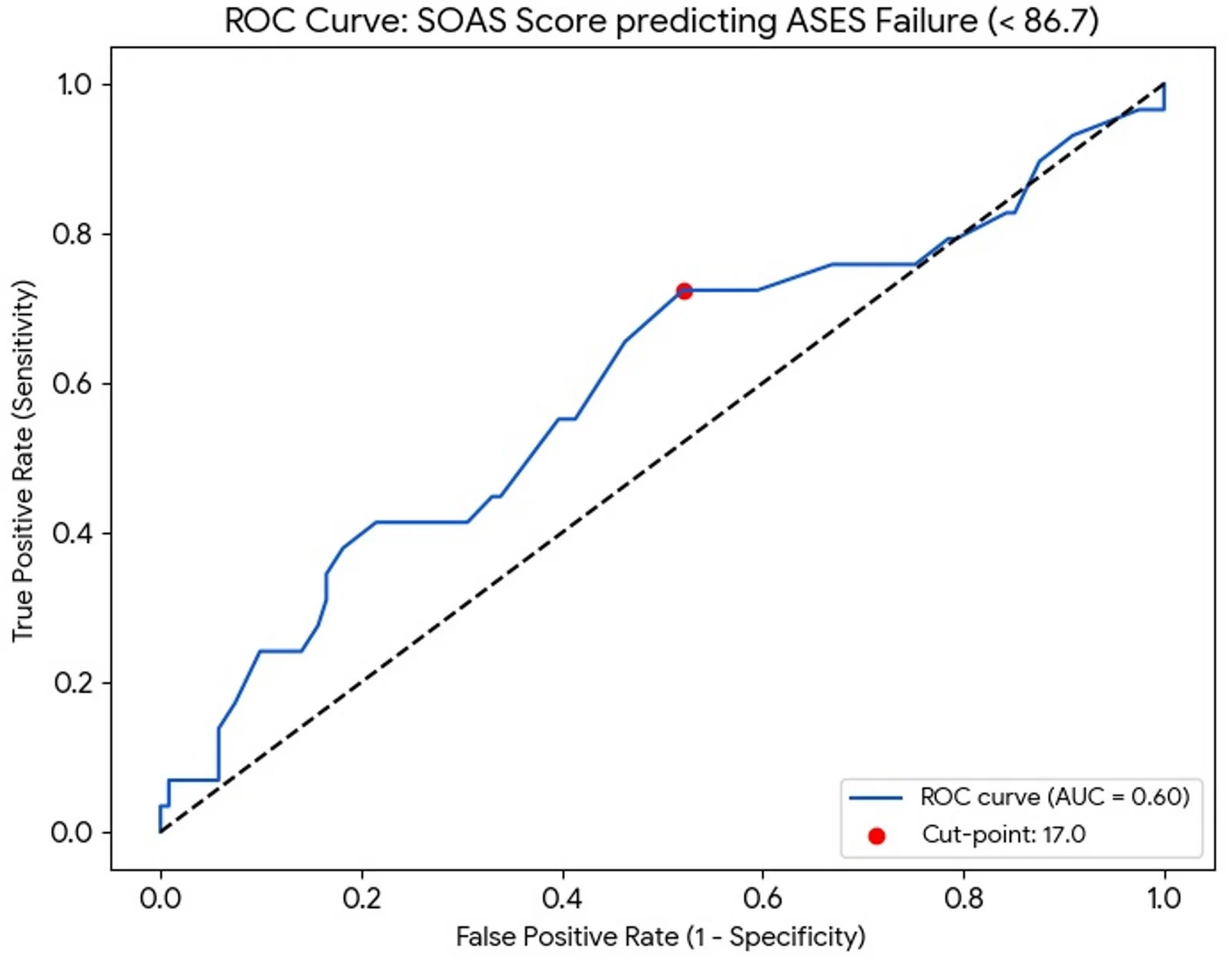
Receiver operating characteristic (ROC) curve of the total SOAS score for predicting postoperative ASES score.

To establish a clinical threshold for surgical decision-making, an ROC curve analysis was performed using the PASS criterion for the ASES score (≥ 86.7). The analysis yielded an optimal SOAS cut-point of 17 (Sensitivity: 72.4%, Specificity: 47.9%). However, the area under the curve (AUC) was 0.596, indicating poor discriminative ability of the SOAS score alone in predicting functional failure following ARCR (Fig. 1).

**Fig. 2:**
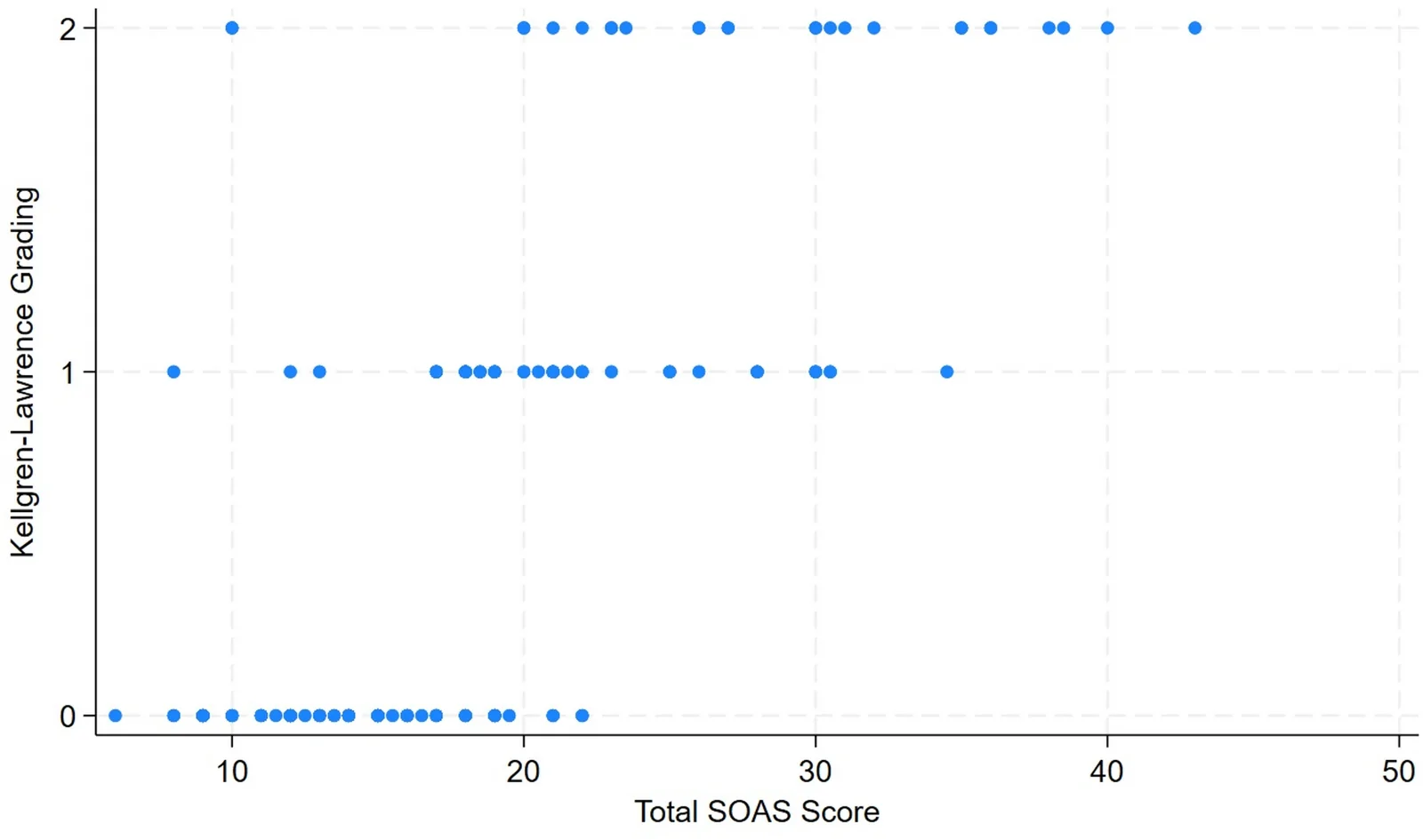
Correlation between MRI-based SOAS score and radiographic Kellgren-Lawrence (KL) grading.

The validity of the SOAS score was confirmed through its correlation with traditional radiographic grading. A strong positive correlation was observed between the total SOAS score and Kellgren-Lawrence grading (*r* = 0.714, *p* < 0.001). Furthermore, the mean SOAS score increased progressively with higher KL grades: 14.09 ± 3.62 for Grade 0, 21.42 ± 5.44 for Grade 1, and 28.21 ± 8.45 for Grade 2 (Fig. 2).

**Fig. 3:**
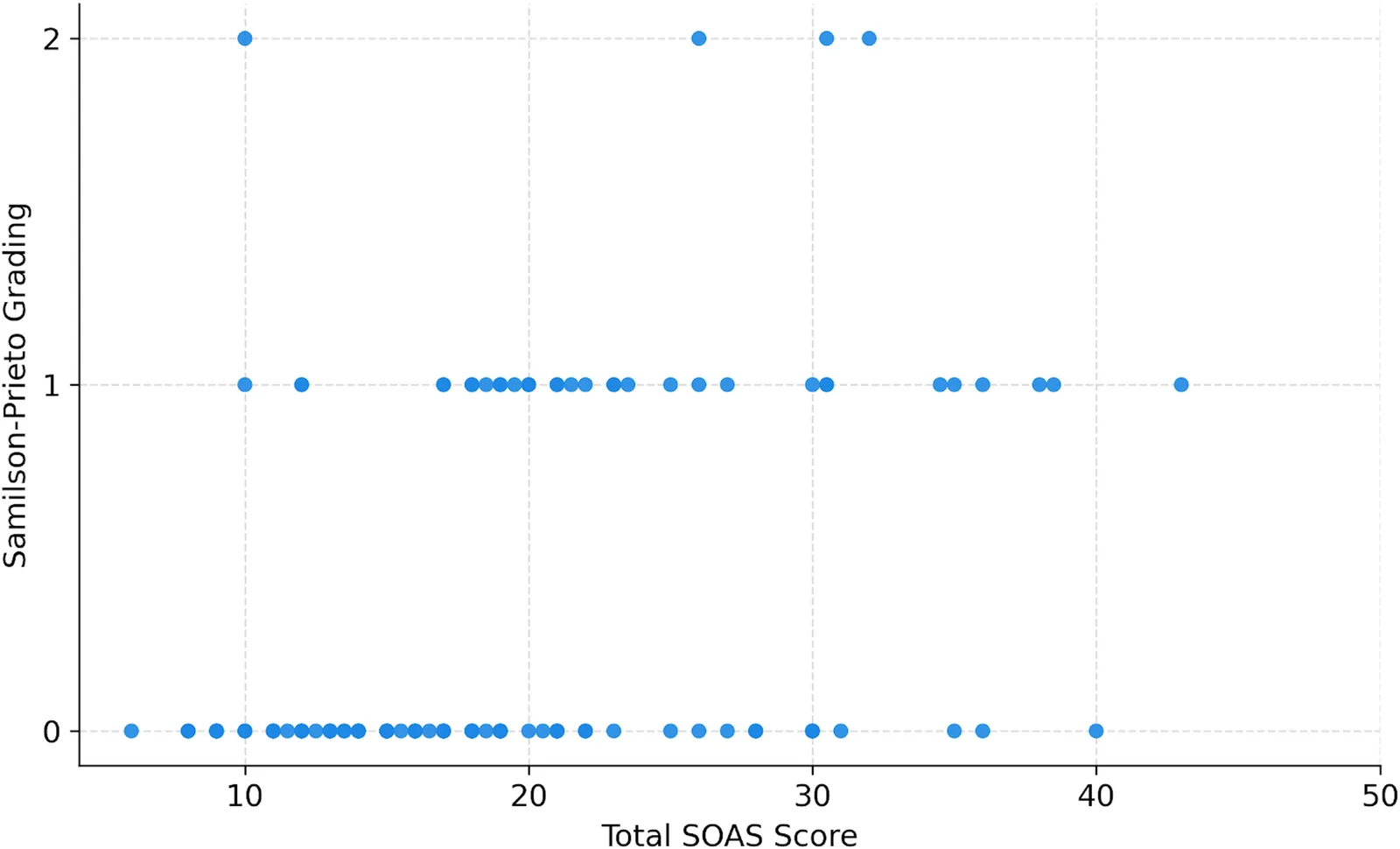
Correlation between MRI-based SOAS score and radiographic Samilson-Prieto grading.

In addition to the KL grading, the Samilson-Prieto grading system was used to assess radiographic osteoarthritis. A moderate positive correlation was observed between the total SOAS score and Samilson-Prieto grading (*r* = 0.401, *p* < 0.001). The mean SOAS scores increased progressively across Samilson-Prieto grades: 16.96 ± 6.46 for Grade 0, 23.91 ± 8.12 for Grade 1, and 24.63 ± 10.08 for Grade 2. (Fig. 3)

## Discussion

This study aimed to determine whether pre-existing GHOA, assessed via the MRI-based SOAS score, independently predicts PROs following ARCR. The most crucial finding demonstrates a nuanced relationship between GHOA severity and postoperative functional outcomes. Our findings demonstrate a consistent relationship between GHOA severity and postoperative functional outcomes. Both the categorical comparison (Table 5), which showed significantly lower ASES scores in patients with SOAS score ≥ 18, and the multivariate regression analysis (Table 4B), which identified the total SOAS score as a significant independent predictor of ASES scores (*p* = 0.011), underscore the clinical impact of pre-existing joint degeneration on ARCR success. Furthermore, specific sub-domain pathologies, notably of the long head of the biceps (LHB), alongside systemic comorbidities such as DM and osteoporosis, were identified as critical independent predictors of clinical outcomes.

Our sub-domain analysis (Table 3) revealed that cartilage defects and acromioclavicular (AC) joint degeneration correlated exclusively with lower UCLA scores, sparing the ASES and WORC indices. This discrepancy can be explained by the structural focus of the respective scoring instruments. The ASES and WORC primarily assess pain and general activities of daily living (ADLs) utilizing mid-range shoulder kinematics. In contrast, the UCLA score inherently incorporates specific evaluations of terminal Active Forward Flexion and Forward Flexion Strength. Biomechanically, glenohumeral cartilage wear generates mechanical friction that limits terminal motion and induces pain-inhibited weakness, directly penalizing the physical performance parameters of the UCLA score. Furthermore, AC joint degeneration is a well-established source of pain specifically during terminal overhead elevation. Consequently, patients with these specific degenerative features may restrict maximal overhead motion—resulting in a lower UCLA score—while remaining capable of performing mid-range ADLs, thereby preserving their ASES and WORC scores.

To further elucidate the clinical utility of the SOAS score, we performed a ROC curve analysis to establish a threshold for predicting failure to achieve the PASS for the ASES score (≥ 86.7)^9^. Although the analysis identified an optimal SOAS cutoff of 17, the AUC was only 0.596 (sensitivity 72.4%, specificity 47.9%). While the SOAS score was identified as an independent predictor of outcomes in the multivariate analysis, its low discriminative ability statistically reinforces that the total severity of joint degeneration, when viewed in isolation, is a poor prognostic discriminator for surgical failure. Consequently, the identified SOAS score cut-off should be interpreted with caution. Ultimately, these findings emphasize that high GHOA severity should not be viewed as an absolute contraindication for surgery; rather, surgical decision-making must integrate other significant clinical predictors.

This discrepancy between categorical and continuous analyses helps reconcile conflicting evidence in the existing literature. Studies utilizing categorical radiographic grading systems, such as Samilson-Prieto, frequently report no significant differences in PROs between patients with and without GHOA, mirroring our categorical findings^23,24^. Conversely, utilizing continuous MRI-based scoring provides a more sensitive evaluation of the degenerative spectrum. Our continuous model findings align with Chi et al.^25^, who demonstrated that an increasing total SOAS score predicted less improvement in ASES scores at 2 years postoperatively. Similarly, Kukkonen et al.^26^ found that incremental increases in osteoarthritis (any KL grade > 0) were associated with significantly lower Constant scores. The SOAS score’s ability to evaluate cartilage integrity, bone morphology, and soft tissue structures concurrently may capture the gradual functional decline that strict categorical cutoffs mask.

Interestingly, our analysis revealed that while the MRI-based SOAS score demonstrated a strong correlation with the KL grading (*r* = 0.714), it exhibited only a moderate correlation with the Samilson-Prieto grading (*r* = 0.401). This discrepancy is clinically sound. The Samilson-Prieto system primarily categorizes severity based on the size of inferior osteophytes, which may not accurately reflect the holistic joint degeneration. Patients with significant cartilage wear or intra-articular pathology—capturable by both MRI (SOAS) and joint space narrowing on radiographs (KL grading)—may present with minimal osteophyte formation, thereby clustering in lower Samilson grades. This highlights the limitation of relying solely on osteophyte-based radiographic grading and underscores the superiority of comprehensive scoring systems like the SOAS score in evaluating the true spectrum of glenohumeral osteoarthritis prior to ARCR.

Beyond overall joint degeneration, our sub-domain analysis highlights that specific intra-articular and systemic factors may drive clinical outcomes more profoundly than generalized wear. First, pathology of the LHB was a significant independent predictor of lower ASES scores. The LHB tendon is densely innervated with a complex network of sensory and sympathetic nerve fibers, making it a well-recognized primary pain generator in the shoulder joint^27^. Severe or unaddressed LHB pathology, such as chronic tenosynovitis or instability, frequently coexists with rotator cuff tears and GHOA, leading to persistent anterior shoulder pain that heavily compromises postoperative patient-reported functional scores. Systemically, DM and osteoporosis strongly predicted inferior PROs across multiple metrics in our cohort. The detrimental impact of DM on rotator cuff healing is fundamentally driven by both biological and microvascular impairments. Chronic hyperglycemia leads to the accumulation of advanced glycation end-products (AGEs) within the tendon extracellular matrix. These AGEs induce abnormal collagen cross-linking, resulting in stiffer, less organized, and biomechanically inferior tendon tissue^28^. Furthermore, DM-induced microangiopathy compromises the delicate vascular network at the rotator cuff footprint, thereby impairing the biological healing process at the tendon-to-bone interface and predisposing the construct to failure regardless of the baseline GHOA. Similarly, osteoporosis directly jeopardizes the mechanical stability of the surgical repair. Diminished bone mineral density and compromised trabecular microarchitecture at the greater tuberosity significantly reduce the pull-out strength of suture anchors^29^. This osteoporotic environment predisposes the repair to mechanical failure at the implant-bone interface before adequate biological healing can occur. Together, these findings strongly suggest that the metabolic impairment of tissue healing (DM) and compromised structural anchor fixation (osteoporosis) dictate the clinical success of ARCR far more profoundly than the concurrent loss of articular cartilage alone.

Importantly, these findings emphasize the critical distinction between the disease and the illness of osteoarthritis. The disease of OA is defined by the pathological changes within joint tissues, whereas the illness of OA encompasses the patient’s subjective experience, including pain, functional limitations, muscle weakness, joint stiffness, and diminished quality of life^30^. Notably, the illness of OA, rather than the disease itself, has been associated with reduced time-to-mortality^31^. Furthermore, pain perception and subsequent functional limitations are influenced by a complex interplay of factors, including genetic predispositions^32,33^, prior experiences^34,35^, current emotional state^36^, coping mechanisms (e.g., catastrophizing)^37^, and socio-cultural context^38–40^. These results suggest that the presence of GHOA alone should not be a contraindication for ARCR.

This study possesses several strengths. The use of the SOAS score, derived from MRI, allowed for a more detailed assessment of glenohumeral osteoarthritis compared to traditional plain radiographs. The SOAS score also demonstrated good inter-rater reliability in our cohort. A significant methodological strength of this study is the novel application of ROC curve analysis to identify a specific clinical cut-point for the SOAS score based on the PASS. This approach successfully bridges the gap between continuous imaging scores and binary clinical decision-making, providing robust statistical evidence that GHOA severity alone has limited predictive utility. Furthermore, the use of multivariate linear regression analysis enabled us to account for potential confounding factors when examining the relationship between GHOA and patient-reported outcomes and. Finally, the sample size calculation (*N* = 150) ensured adequate statistical power to detect clinically meaningful differences. Despite these strengths, several limitations must be acknowledged, which are inherent to the study design and data collection methods. The retrospective design introduces potential selection bias. The follow-up period, ranging from two to five years post-operatively, may not be sufficient to capture long-term changes in joint degeneration and functional outcomes. Furthermore, it is important to acknowledge that while the reported correlations are statistically significant, their magnitude is relatively weak. Consequently, these specific associations possess a modest and limited standalone clinical impact. Rather than serving as isolated prognostic indicators, they should be interpreted as contributing elements within a broader, multifactorial clinical picture when evaluating patient outcomes. Another notable limitation of the current study is the absence of preoperative baseline functional outcome scores. Because these baseline metrics were not available, our analysis is strictly confined to evaluating the final postoperative functional status, rather than quantifying the magnitude of clinical improvement following ARCR. Consequently, we cannot determine whether patients with advanced GHOA experienced a smaller overall trajectory of recovery compared to those without. While this is a limitation, numerous previous studies support the notion that ARCR can significantly improve PROs from pre-operative levels^41^. The use of standardized telephone interviews may have introduced some variability in data collection due to differences in questioning and patient interpretation. The fact that surgical procedures were performed by five different surgeons also introduces the potential for variability in surgical technique and decision- making. Finally, despite our efforts to collect data on a comprehensive set of factors, there may be other unmeasured confounders, such as psychological factors or social support, that could have influenced patient-reported outcomes.

Future prospective, multi-center longitudinal studies incorporating pre-operative baseline scores are necessary to refine the prognostic value of the SOAS score. Additionally, qualitative assessments of the patient experience and economic evaluations comparing ARCR to arthroplasty in the setting of GHOA will be vital for optimizing clinical decision-making.

## Conclusion

The preoperative MRI-based SOAS score is an independent predictor of inferior functional outcomes following arthroscopic rotator cuff repair. However, the use of a strict SOAS cutoff demonstrates poor discriminative ability for predicting clinical failure and should not be considered an absolute contraindication for rotator cuff repair. Our findings highlight that surgical success is more profoundly dictated by specific intra-articular pain generators, notably long head of the biceps pathology, and systemic metabolic compromises, particularly diabetes mellitus and osteoporosis. To optimize clinical decision-making and manage patient expectations effectively, clinicians must adopt a comprehensive preoperative risk stratification approach that integrates these critical structural and systemic factors rather than relying solely on the severity of joint degeneration.

## Data Availability

The datasets generated and/or analyzed during the current study are available from the corresponding author on reasonable request.

## Abbreviations

ARCR: Arthroscopic rotator cuff repair
GHOA: Glenohumeral osteoarthritis
SOAS: Shoulder osteoarthritis severity score
PROs: Postoperative patient-reported outcomes
MRI: Magnetic resonance imaging
ASES: American shoulder and elbow surgeons score
UCLA: University of california-los angeles score
WORC: Western ontario rotator cuff index
DM: Diabetes mellitus
BMI: Body mass index
KL: The kellgren and lawrence grading
ROC: Receiver operating characteristic
PASS: Patient acceptable symptom state
MCID: Minimally clinically important difference
ICC: Intraclass correlation coefficient
